# Decreased Chromosomal Damage in Lymphocytes of Obese Patients After Bariatric Surgery

**DOI:** 10.1038/s41598-018-29581-6

**Published:** 2018-07-25

**Authors:** Ezgi Eyluel Bankoglu, Charlotte Arnold, Ilona Hering, Mohammed Hankir, Florian Seyfried, Helga Stopper

**Affiliations:** 1 Institute of Pharmacology and Toxicology, University of Wuerzburg, Wuerzburg, Bavaria Germany; 2 Department of General, Visceral, Vascular and Pediatric Surgery, University Hospital of Wuerzburg, Wuerzburg, Bavaria Germany; 3 Experimental Surgery, Department of General, Visceral, Vascular, and Pediatric Surgery, University Hospital of Wuerzburg, Wuerzburg, Bavaria Germany

## Abstract

The number of bariatric surgeries being performed worldwide has markedly risen. While the improvement in obesity-associated comorbidities after bariatric surgery is well-established, very little is known about its impact on cancer risk. The peripheral lymphocyte micronucleus test is a widely used method for the monitoring of chromosomal damage levels *in vivo*, and micronucleus frequency positively correlates with cancer risk. Therefore, the aim of this study was to compare the micronucleus frequency before and after bariatric surgery in obese subjects. Peripheral blood mononuclear cells were collected from 45 obese subjects before and at two time-points after bariatric surgery (6 and 12 months) to assess spontaneous micronucleus frequency. Consistent with the increased cancer risk previously shown, bariatric surgery-induced weight loss led to a significant reduction in lymphocyte micronucleus frequency after 12 months. Interestingly, comorbidities such as type 2 diabetes mellitus and metabolic syndrome further seemed to have an impact on the lymphocyte micronucleus frequency. Our findings may indicate a successful reduction of cancer risk in patients following weight loss caused by bariatric surgery.

## Introduction

Obesity is one of the most pressing health challenges of the 21st century. According to the WHO, over 600 million adults around the world suffer from obesity, which is closely associated with other chronic diseases such as type 2 diabetes mellitus (T2DM), hypertension, non-alcoholic fatty liver disease (NAFLD) and cardiovascular disease^1^. The clustering of obesity, insulin resistance and dyslipidemia in individuals has been referred to as metabolic syndrome^2^. There are reports of increased cancer risk among obese individuals, with T2DM itself representing an independent risk factor. Since 80% of T2DM patients are overweight or obese, cancer risk becomes even more important for the subgroup of patients with metabolic syndrome^3^.

Conservative weight loss regimens are limited in their efficiency, making bariatric surgery the superior therapeutic option - especially for morbid obesity and related comorbidities^4,5^. Indeed, various follow-up studies have demonstrated the successful remission of T2DM associated with bariatric surgery-induced weight loss as well as a reduction in mortality^6–8^. Similarly, the Swedish Obese Subjects (SOS) study and the Utah Cancer Registry (UCR) reported a decrease in cancer-related mortality after bariatric surgery^8–10^. However, elevated (colon) cancer incidences have also been reported^11^.

Genomic instability is one of the driving forces for carcinogenesis. Micronuclei, chromatin containing structures in the cytoplasm, are a good marker of genomic damage and are a subtype of chromosomal aberrations^12,13^. The micronucleus test is a well-established method for the detection of genotoxicity. For human biomonitoring, peripheral blood-derived lymphocytes are the most often used cells^14,15^. Numerous studies have reported increased micronucleus frequency in peripheral blood-derived lymphocytes among cancer patients and population studies indicated a correlation between cancer risk and micronucleus frequency, supporting a positive association with cancer risk^16–24^. An increase of micronuclei has been found in obesity^25–28^, but the impact of weight loss on genomic damage has not been investigated so far.

Therefore, the purpose of this study was to analyze the possible influence of weight loss induced by bariatric surgery on chromosomal damage as detected by micronucleus formation.

## Materials and Methods

### Materials

Unless stated otherwise, chemicals were purchased from Sigma Aldrich Germany (Munich, Germany). Cell culture media and reagents were purchased from PAA Laboratories (Pasching, Austria) and Life Technologies (Darmstadt, Germany). Fetal bovine serum (FBS) was from Biochrom (Berlin, Germany). Microscope slides were from Asisstent (Sondheim, Germany) and methanol was from thgeyer (Renningen, Germany). Gel Green was purchased from Biotrend (Köln, Germany).

### Methods

#### Studied Subjects

Blood samples were collected from 45 morbidly obese (38 female and 7 male) subjects before, 6 and 12 months after the bariatric surgery. The bariatric surgery was performed in the Surgery Department, University Hospital Wuerzburg. Sample collection was conducted in the Surgery and Endocrinology Departments, University Hospital Wuerzburg and all participants gave written informed consent. This study was approved by the Ethics Committee of the University of Wuerzburg (Study No: 186/14). All experiments were performed in accordance with relevant guidelines and regulations.

Insulin resistance had been determined prior to this study by HOMA-IR (homeostasis model assessment-insulin resistance) and the presence of a metabolic syndrome was defined according to the current definition of the international diabetes federation consensus worldwide definition of the metabolic syndrome^29^.

#### Blood sampling and isolation of mononuclear cells

Blood samples were obtained from 45 obese subjects with a BMI of 51.02 ± 0.94 kg/m^2^ and an age of 44.37 ± 1.62 years. Sampling was performed before and after (about 6 and 12 months) bariatric surgery. Whole blood was collected into commercially available EDTA tubes. Peripheral blood mononuclear cells (PBMCs) were isolated by density gradient centrifugation on histopaque. Blood samples were layered on histopaque surface (1:1) and were centrifuged at room temperature 1600rpm for 30 min. After centrifugation, the so-called “buffy coat” containing mononuclear cells was pipetted into a 15 ml tube and washed two times with RPMI 1640 medium (containing 1% FBS, 1% L-glutamine and penicillin) at 12000 rpm for 10 min at room temperature (RT). Isolated PBMCs were used for micronucleus assessment. Blood parameters were determined by the central laboratory of the University Hospital Wuerzburg.

#### Cytokinesis block micronucleus assay

After isolation, PBMCs were washed two times with RPMI medium (containing 1% FBS, 1% L-glutamine and penicillin) and cell number was determined by a cell counting chamber. 1 × 10^6^ cells/ml were cultivated with 1 µg/ml phytohaemagglutinin (PHA) for 44 hours at 37 °C with 5% CO_2_. Subsequently, 3 µg/ml cytochalasin B was added for another 24 hours to yield binucleated cells. On the next day, cell number was determined and cells were diluted to 300,000 cells/ml. One hundred µl of this cell suspension was placed on to a microscope slide (2 slides for each patient 30,000 cells/slide) by cytospin centrifugation (at 1000 rpm for 5 min) and fixed in methanol at −20 °C for at least two hours.

Shortly before staining, two slides for each patient were air dried under the fume hood and stained with gel green (1:100 diluted in bidistilled water) and mounted with Dabco (1, 4- diazabicyclo[2.2.2]octane) for microscopic evaluation. For each patient, two replicate slides and 1000 binucleated cells per slide were scored for micronuclei. As a quality criteria of the lymphocyte culture, proliferation index was assessed. 1000 cells from two replicate slides were scored for the assessment of the proliferation index (PI) for the categories of mono- (MoN), bi- (BN) and multi-nucleated (MuN) cells together with apoptosis, mitosis, nucleoplasmic bridges and nuclear buds^15^. The calculation of the PI was done according to the following formula. The frequency of apoptosis and mitosis were represented as per mill (%_o_). The frequency of nucleoplasmic bridges and nuclear buds were less than 1% (data not shown).

$$PI=\frac{(1\,\times \,MoN)+(2\,\times \,BN)+(3\,\times \,MuN)}{(MoN+BN+MuN)}$$

### Statistics

Statistical analysis was performed using SPSS 22 software. Data are presented as Mean ± SEM. Normality of the data was checked with the Shapiro-Wilk test and according to the results either a t-test or the Wilcoxon test (for independent samples Mann-Whitney) was performed to check for significant differences between individual groups. Results were considered significant with p ≤ 0.05.

### Data availability

The datasets generated during the current study are available from the corresponding author on reasonable request.

## Results

### Patient blood biochemistry, comorbidities and weight loss

**Figure 1:**
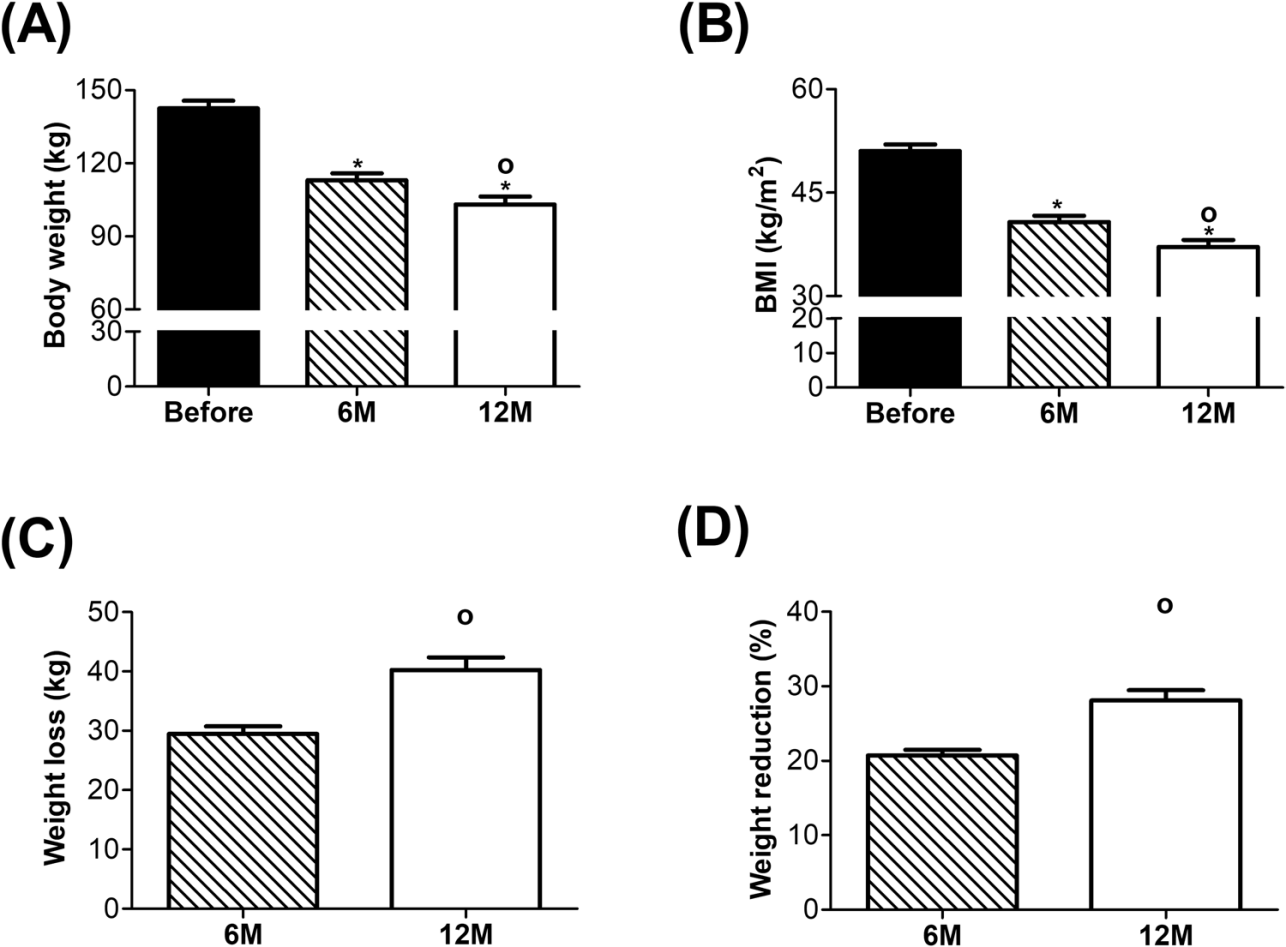
Body weight, BMI and weight loss before and after bariatric surgery. (**A**) Body weight in kg, (**B**) Body Mass Index (BMI kg/m^2^), (**C**) Weight loss in kg and (**D**) Weight reduction in %. Before surgery, n = 45; 6 M, n = 35; 12 M, n = 45. *p ≤ 0.05 significant vs. Before and °p ≤ 0.05 significant vs. 6 M.

At baseline, obese study participants were at an average age of 44.37 ± 1.62 years with a BMI of 51.02 ± 0.94 kg/m^2^. Six months after surgery, obese subjects lost a significant amount of excess body weight and reached an average BMI of 40.76 ± 0.92 kg/m^2^ (Fig. 1A,B). In the second 6-month period, subjects continued to lose a significant amount of excess body weight and reached an average BMI of 37.11 ± 1.04 kg/m^2^ by the end of our study Weight loss in kg and weight reduction as percent of previous body weight are shown in Fig. 1C,D, respectively. A summary for the body weight (kg), BMI, and age over the experimental time frame as well as gender distribution can be seen in Table 1.

**Table 1:** Summary for body weight, BMI, age and gender before and after surgery.

Groups	Body weight (kg)	BMI (kg/m^2^)	Age	Gender
Before	142.55 ± 3.07	51.02 ± 0.94	44.37 ± 1.62	Female: 38 Male: 7
6 M	113.06 ± 2.74	40.76 ± 0.92	—	—
12 M	103.14 ± 3.09	37.11 ± 1.04	—	—

Elevated levels of some important biochemical blood parameters were found in morbidly obese subjects before surgery and, as soon as 6 months afterwards, there were significant improvements that generally remained so for the duration of the 12-month recording period. Among these parameters, a progressive reduction in CRP levels indicated the improvement of the chronic inflammation status and the significantly reduced glycated haemoglobin and fasting glucose levels (HbA1c) pointed to the improvement of glucose homeostasis. Notably, LDL and HDL levels showed a reciprocal pattern progressively decreasing and increasing after surgery, respectively, consistent with an improvement in lipid homeostasis. Precise biochemical blood parameters of the study subjects can be seen in Table 2 and distribution of related comorbidities is shown in Table 3.

**Table 2:** Biochemical blood parameters.

Parameters	Before	After 6 months	After 12 months	Significance
Glucose (mg/dl)	99.14 ± 3.90 (N = 44)	86.52 ± 3.61 (N = 44)	94.50 ± 5.22 (N = 42)	^*,#^
Alkaline phosphatase (U/l)	80.14 ± 5.41 (N = 44)	82.07 ± 4.06 (N = 44)	75.60 ± 3.53 (N = 42)	^∆^
Triglyceride (mg/dl)	139.61 ± 10.44 (N = 33)	110.82 ± 5.50 (N = 44)	105.24 ± 7.22 (N = 42)	^*,#^
Cholesterol (mg/dl)	190.12 ± 7.11 (N = 33)	180.27 ± 6.26 (N = 44)	178.93 ± 6.73 (N = 42)	^*,#^
LDL (mg/dl)	114.15 ± 6.84 (N = 33)	105.93 ± 5.75 (N = 44)	97.67 ± 6.09 (N = 42)	^*,#,∆^
HDL (mg/dl)	48.12 ± 9.86 (N = 33)	52.23 ± 1.56 (N = 44)	59.62 ± 2.07 (N = 42)	^#,∆^
CRP (mg/dl)	1.18 ± 0.18 (N = 45)	0.59 ± 0.09 (N = 44)	0.54 ± 0.11 (N = 42)	^*,#,∆^
HbA1c (%)	5.94 ± 0.18 (N = 36)	5.42 ± 0.08 (N = 45)	5.45 ± 0.11 (N = 42)	^*,#^

LDL: low density lipoprotein, HDL: high density lipoprotein, CRP: C-reactive protein and HbA1c: glycated hemoglobin *p ≤ 0.05 Before vs. After 6 months, ^#^p ≤ 0.05 Before vs. After 12 months and ^∆^p ≤ 0.05 After 6 months vs. After 12 months.

**Table 3:** Comorbidities before surgery.

Comorbidities	Number	%
IR	14/45	31.1
T2DM	13/45	28.9
IDDM	6/45	13.3
NASH/NAFLD	40/45	88.9
Hypertension	30/45	66.7
Metabolic syndrome	14/45	31.1

IR: insulin resistance, T2DM: type 2 diabetes mellitus, IDDM: insulin dependent diabetes mellitus, NASH: non-alcoholic steatohepatitis, NAFLD: non-alcoholic fatty liver disease.

### Analysis of lymphocyte micronucleus frequency

**Figure 2:**
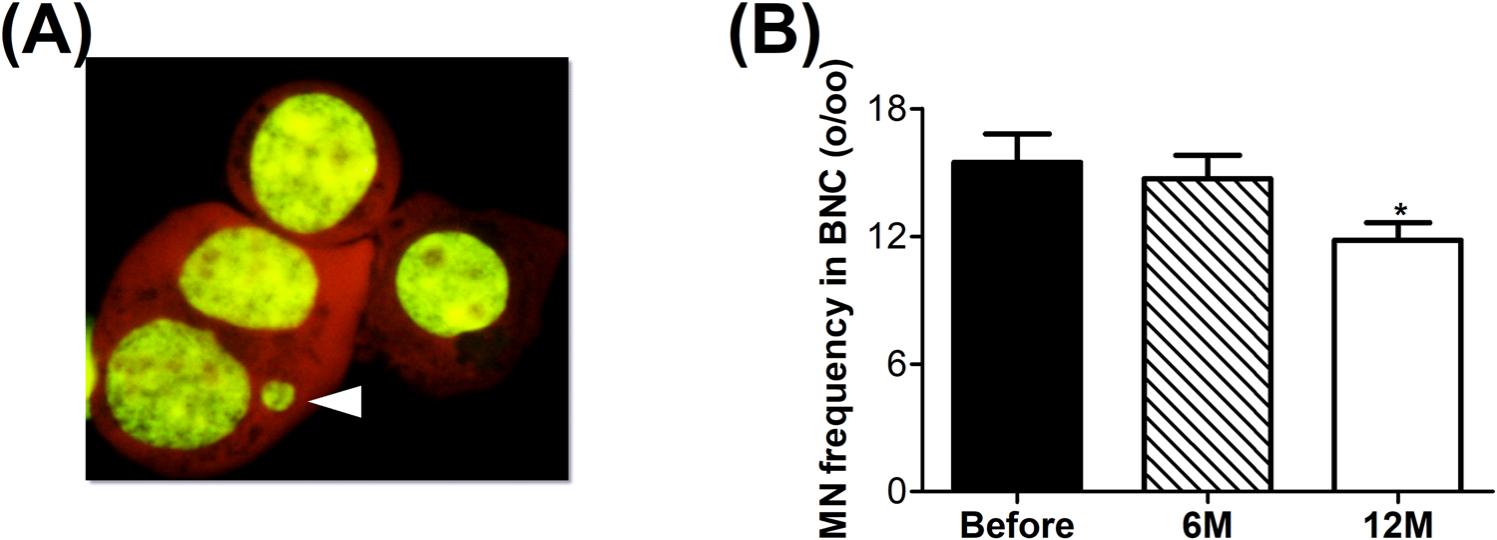
Micronucleus frequency per mill (%_o_) in isolated lymphocytes of obese subjects. (**A**) Representative picture of a micronucleus in a binucleated cell indicated by the white arrow. (**B**) Micronucleus frequency per mill (%_o_) before and after surgery. Before, n = 45; 6 M, n = 35 and 12 M, n = 45. *p ≤ 0.05 significant vs. before.

The cytokinesis blocking micronucleus test was used to determine the spontaneously accumulated chromosomal/genomic damage in lymphocytes. A representative picture of a micronucleus in a binucleated cell (BNC) can be seen in Fig. 2A. Six months after surgery, there was no significant difference in micronucleus number of obese subjects compared to before surgery. However, a significant reduction was observed 12 months after surgery (Fig. 2B). In Table 4, proliferation index, mitosis and apoptosis per mill can be found. After the weight loss a significant increase in proliferation index and mitosis were observed with a significant reduction in apoptosis.

**Table 4:** Proliferation Index (PI), Mitosis and Apoptosis.

Groups	PI	Mitosis (%_o_)	Apoptosis (%_o_)
Before	1.52 ± 0.03	12.87 ± 1.34	9.60 ± 1.47
6 M	1.60 ± 0.03	20.81 ± 1.74 (*)	6.77 ± 1.02
12 M	1.59 ± 0.03 (#)	18.88 ± 1.23 (#)	5.63 ± 1.21 (#)

^*^p ≤ 0.05 Before vs. After 6 months and ^#^p ≤ 0.05 Before vs. After 12 months.

**Figure 3:**
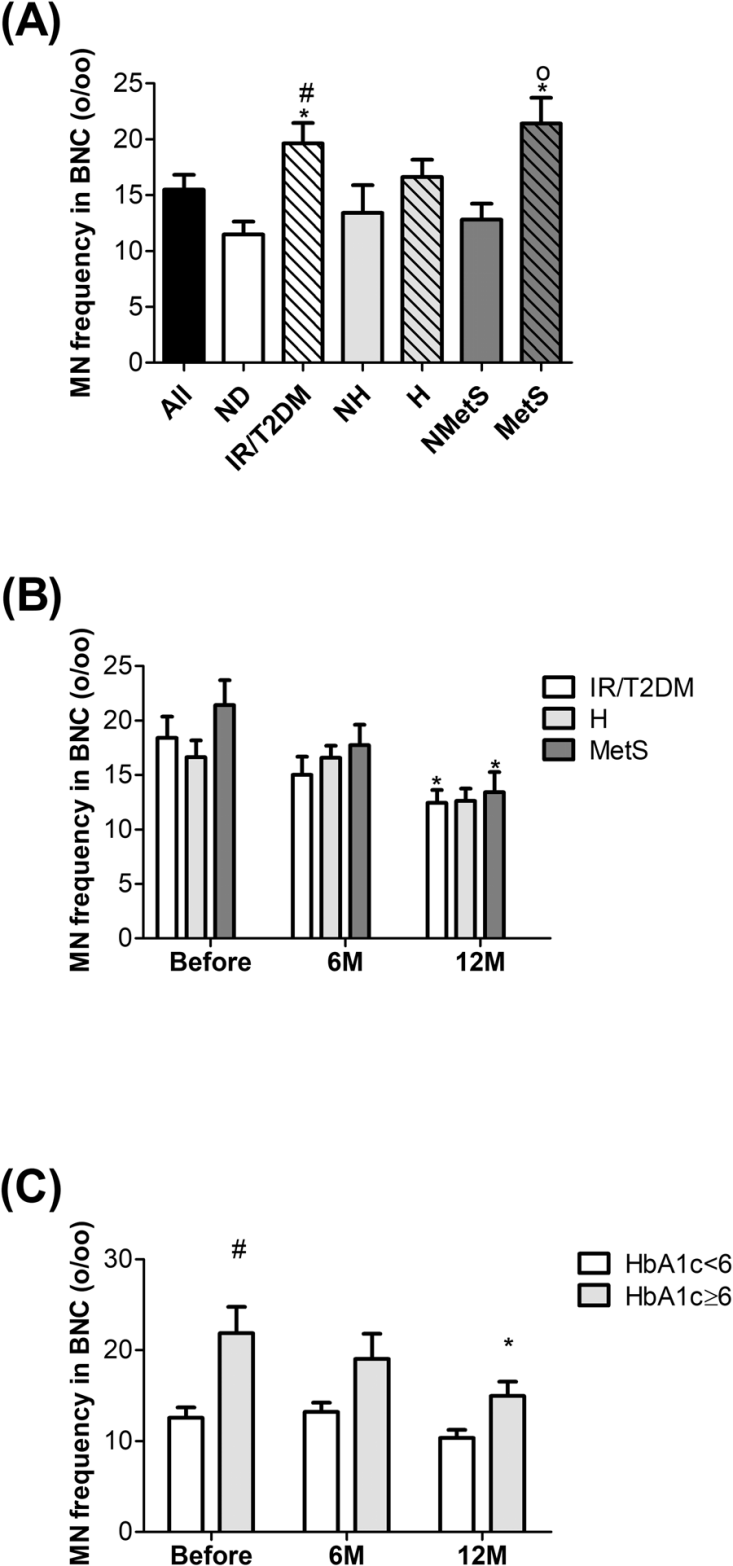
Micronucleus frequency per mill (%_o_) among the subgroups of obese subjects before surgery. (**A**) Comparison of micronucleus frequency between all patients, nondiabetic (ND), IR/T2DM, nonhypertensive (NH), hypertensive (H), nonmetabolic syndrome (NMetS) and metabolic syndrome (MetS). (**B**) Comparison of micronucleus frequency between IR/T2DM, hypertensive (H) and metabolic syndrome (MetS) before and after surgery. (**C**) Comparison of micronucleus frequency between a subgroup of HbA1c < 6 and HbA1c ≥ 6 before and after surgery. Before surgery, n_All_ = 45, n_ND_ = 19, n_IR/T2DM_ = 26, n_NH_ = 16, n_H_ = 29, n_NMetS_ = 31 and n_MetS_ = 14; 6 M, n_IR/T2DM_ = 21, n_H_ = 21, and n_MetS_ = 10; 12 M, n_IR/T2DM_ = 26, n_H_ = 29 and n_MetS_ = 14. *p ≤ 0.05 significant vs. All or vs. Before_IR/T2DM_ or vs. Before_MetS_, ^#^p ≤ 0.05 significant vs. ND or vs. Before_HbA1c≥6_ and op ≤ 0.05 significant vs. NMetS.

After dividing patients into subgroups according to comorbidities (insulin resistance (IR)/type 2 diabetes mellitus (T2DM), hypertension (H) and metabolic syndrome (MetS)), significant differences in micronucleus frequencies were detected (Fig. 3). The insulin resistant/T2DM group showed a significantly higher micronucleus frequency compared to the nondiabetic (ND) group. The hypertensive (H) subgroup did not show any significant difference compared to the non-hypertensive (NH) subgroup. The metabolic syndrome subgroup had a significantly higher micronucleus number compared to patients not affected by metabolic syndrome (Fig. 3A). Within these subgroups that are displayed in Fig. 3A, the small group of nondiabetic/non-hypertensive patients (n = 4) exhibited the lowest micronucleus frequency with 8.87 ± 2.81 (mean MN/1000 BNC ± SEM) and another small group of patients with insulin-dependent diabetes mellitus (n = 5) showed the highest micronucleus frequency with 25.82 ± 4.40 (mean MN/1000 BNC ± SEM) among all groups before surgery.

In Fig. 3B, the micronucleus frequency of subgroups with IR/T2DM, hypertension and metabolic syndrome are shown before and after weight loss. Within the IR/T2DM group, we observed a significant reduction in micronucleus frequency after 12 months. Hypertensive patients did not show a significant difference. Patients with metabolic syndrome (obese, IR or T2DM and hypertensive) showed significant reduction in micronucleus number at 12 months after surgery.

Since our observation showed a strong effect in patients with metabolic syndrome and IR/T2DM, but not with hypertension, we decided to have a closer look on the effect of blood glucose levels on micronucleus formation. Therefore, glycated haemoglobin (HbA1c) was taken as a separate criteria and patients before and after surgery were divided into two groups according to their HbA1c levels (Fig. 3C). The patients with HbA1c levels ≥6 showed significantly higher micronucleus numbers than the patients with HbA1c levels <6 mg/dl. Only the patients with HbA1c levels ≥6 showed a significant reduction in micronucleus number 12 months after the surgery.

## Discussion

Increased DNA damage in obese subjects compared to healthy controls has consistently been documented^25–28^.

In a previous study performed on obese Zucker rats, we found that increased genomic damage in kidney, liver and colon was improved after weight loss induced either by gastric bypass surgery or by chronic caloric restriction^30^. This prompted us to observe the genomic damage level of an obese patient group before and at two time-points during the first year after bariatric surgery. There was a clear and significant reduction in micronucleus frequency after 12 months but not after 6 months. The significant increase in proliferation index and mitosis with a significant reduction in apoptosis indicated improved fitness of the isolated lymphocytes after weight loss. It has to be kept in mind that even 12 months after bariatric surgery, patients still classify as severely obese.

To our knowledge, this is the first study in which genomic damage after bariatric surgery has been analyzed. Two previously published clinical studies did however demonstrate an effect of weight loss induced by bariatric surgery on oxidative stress. Billeter and colleagues^31^ demonstrated a significant reduction of insulin resistance, leptin values, inflammation and oxidative stress 24 months after bariatric surgery. Horn and colleagues^32^ also showed reduced oxidative stress in 16 obese women 180 days after bariatric surgery. The average BMI of the participants was 44.1 ± 6.8 kg/m^2^ before surgery. Even though their starting BMI was lower than that of our obese group (BMI 51.02 ± 0.94 kg/m^2^), the significantly reduced lipid peroxidation and carbonylated protein level of the patients after surgery were still significantly higher than those of the healthy controls. Also, while weight loss may reduce the chronic low inflammatory status of obese patients, the dramatic reduction of fat tissue may burden the body with oxidative stress-inducing molecules. Thus, the situation may be complex and may depend on the amount of fat lost and the starting BMI. At the time point of 6 months (i.e. about 180 days), our patients exhibited significantly lowered CRP (reflecting reduced inflammation), which is in accordance with the published findings on oxidative stress. However, CRP was slightly higher than the healthy reference range. In future studies, a comparison between different extents of obesity might be helpful to see whether there is threshold of final weight or amount of weight loss for reduction of the genomic damage or oxidative stress markers to a healthy control level. It would be further of interest to determine whether a chronic caloric restriction-induced weight loss without surgery can lead to reductions in DNA damage.

In addition to the excess body weight, there are several other health concerns associated with obesity including cardiovascular disease, type 2 diabetes mellitus, hypertension and non-alcoholic fatty liver disease. All these comorbidities are known for their association with oxidative stress^33–37^. Increased oxidative stress could damage cellular macromolecules including DNA. The most common comorbidities among our obese subjects were non-alcoholic fatty liver disease together with steatohepatitis, hypertension and T2DM/Insulin resistance. We divided the obese subjects according to some of these comorbidities and compared the micronucleus frequency among these subgroups. Since the vast majority (89%) of the subjects had either non-alcoholic fatty liver disease (NAFLD) or non-alcoholic steatohepatitis (NASH), we did not use these two comorbidities as an extra category. Patients with hypertension and T2DM/insulin resistance (IR) were referred to as metabolic syndrome subgroup. Before bariatric surgery, the T2DM/insulin resistance (IR) subgroup showed a significantly higher micronucleus frequency compared to the remaining groups. Within the diabetic patients, the small subgroup of insulin dependent patients showed a significantly higher micronucleus frequency. This is consistent with previous findings that T2DM in itself is a risk factor for cancer (3). The reduction of genomic damage in T2DM/IR patients after bariatric surgery might indicate a direct effect of insulin which we have previously shown *in vitro* and in rodent models *in vivo*^38–40^. The putative mechanism behind this might be that increased insulin levels cause an additional increase of ROS formation and ROS induced DNA damage via mitochondrial or NADPH oxidase activation^41,42^.

Interestingly, hypertension alone did not lead to any significant elevation of micronucleus formation. Nevertheless, the metabolic syndrome subgroup showed a significant increase in micronucleus frequency. This points to a synergistic effect of hypertension.

In summary, the findings from the present study support the existing knowledge about increased DNA damage in obese subjects and for the first time demonstrate the beneficial effect of bariatric surgery-induced weight loss on micronucleus frequency in humans. However, there are still some important knowledge gaps, such as whether a reduction to the genomic damage level of healthy individuals can be achieved over a longer time after surgery or with a more extensive weight loss, and whether particular methods of weight loss are superior to others. In addition, a comparison of MN frequency from volunteers with a wide range of BMI would be interesting though there was no significant correlation between MN frequency and BMI in this study. In addition to all other beneficial health improvements in severely obese patients, bariatric surgery induced weight loss lowers the genomic damage and this may represent a lower cancer risk.
